# Fournier's Gangrene due to Masturbation in an Otherwise Healthy Male

**DOI:** 10.1155/2012/154025

**Published:** 2012-05-22

**Authors:** Jason D. Heiner, Katisha D. Eng, Todd A. Bialowas, Diane Devita

**Affiliations:** ^1^Department of Emergency Medicine, Brooke Army Medical Center, Fort Sam Houston, San Antonio, TX 78234, USA; ^2^Department of Emergency Medicine, Madigan Army Medical Center, Fort Lewis, Tacoma, WA 98431, USA

## Abstract

Fournier's gangrene is a rare and often fulminant necrotizing fasciitis of the perineum and genital region frequently due to a synergistic polymicrobial infection. This truly emergent condition is typically seen in elderly, diabetic, or otherwise immune-compromised individuals. Here, we report an unusual case of Fournier's gangrene due to excessive masturbation in an otherwise healthy 29-year-old male who presented to the emergency department complaining of two days of fever, vomiting, and diffuse myalgias. Upon further questioning, he also endorsed severe scrotal pain and swelling and frequent masturbation with soap as a lubricant resulting in recurrent penile erythema and minor skin abrasions. Examination of the patient's perineum was consistent with Fournier's gangrene and included significant erythema, edema, and calor of the penis and scrotum with a large malodorous eschar. He was given intravenous antibiotics and immunoglobulin and promptly underwent three surgical debridements of the scrotum and penis with split-thickness skin grafting. Complications from excessive masturbation are exceedingly rare, but as this case illustrates, they can be life threatening.

## 1. Introduction

Fournier's gangrene is a rare and often fulminant necrotizing fasciitis of the perineum and genital region frequently due to a synergistic polymicrobial infection [1–5]. This truly emergent condition is typically seen in elderly, diabetic, or otherwise immune compromised individuals [1–5]. Here, we report an unusual case of Fournier's gangrene due to excessive masturbation in an otherwise healthy 29-year-old male.

## 2. Case Presentation

An otherwise healthy 29-year-old male presented to the emergency department (ED) after being evaluated at an outside urgent care clinic for two days of fever, vomiting, and diffuse myalgias. Upon further questioning, he also endorsed severe scrotal pain and swelling and frequent masturbation with soap as a lubricant. He reported that past episodes of masturbation often resulted in recurrent penile erythema and abrasions which had worsened over the previous three days since his last masturbation episode. He denied any recent travel, notable lapses in personal hygiene, or any other preceding genitourinary injury or symptoms.

**Figure 1:**
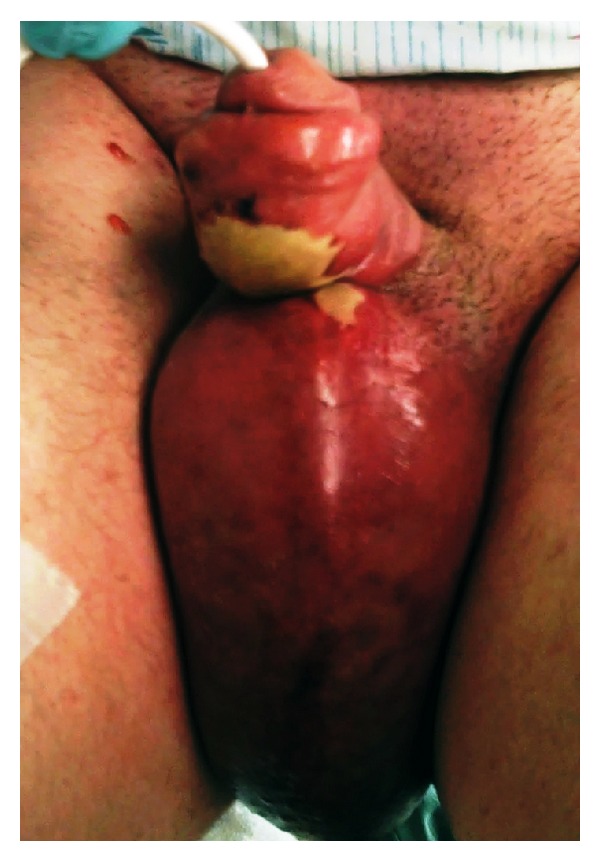
Appearance of the patient's perineum upon emergency department presentation.

The patient appeared alert but ill and in pain, with rigors and a rectal temperature of 104.3°F. His initial blood pressure was 87/50 mmHg, heart rate was 124 beats/min, and respiratory rate was 24 breaths/min with an oxygen saturation of 100% on room air. His physical exam was remarkable for significant erythema, edema, and calor of the penis and scrotum extending to the region of the pubis symphysis but sparing the glans (Figure 1). A large malodorous eschar was noted to the ventral surface of the penis. Aggressive intravenous (IV) fluid resuscitation with normal saline was begun and, with a provisional clinical diagnosis of Fournier's gangrene, IV clindamycin, and ampicillin/sulbactam, was administered, and surgery was consulted. His initial ED labs were remarkable for a white blood cell count of 12,000/mm^3^ and a lactate of 2.2 mEq/L.

The patient was taken to the operating room where cystoscopy and anoscopy were found to be normal with no sign of gangrenous extension or source of infection from the bladder or rectum. He underwent three separate surgical debridements of the scrotum and penis as well as penoscrotal split-thickness skin grafting. Intravenous immunoglobulin (IVIG) was added to his treatment regimen and blood cultures identified strains of *Staphylococcus aureus* and *Streptococcus pyogenes*. On hospital day 22, he was discharged home.

## 3. Discussion

In the late 1800s, the Parisian dermatologist and venereologist Professor Jean-Alfred Fournier used the term “fulminant gangrene” of the penis and scrotum to describe a sudden onset of rapidly progressing idiopathic scrotal gangrene in young men [6]. Today, this rare and often fulminant necrotizing fasciitis of the perineum and genital region is typically seen in elderly, diabetic, or otherwise immune-compromised individuals (with a male predominance) and is known to be frequently due to a synergistic polymicrobial infection [1–5]. The nidus of infection is typically urogenital or anorectal, but cutaneous sources of infection have been reported, with poor personal hygiene acting as an apparent contributing element of infection occurrence [1–5]. Reported mortality rates range from 3% to 45%, affected by factors such as underlying comorbidities, the source of infection, and the presence of severe illness or sepsis upon initial evaluation and treatment [1–5]. Consultation for early surgical debridement and initiation of broad-spectrum IV antibiotics to cover Gram-positive, Gam-negative, and anaerobic bacteria is critical, and the addition of other adjunctive therapies such as IVIG and hyperbaric oxygen therapy may be considered [1–5, 7–9].

While the occurrence of Fournier's gangrene in an otherwise healthy young adult is unanticipated in modern times, frequent masturbation as the underlying cause of this condition is even more unexpected. An extensive review of the current medical literature rarely reveals past reports of Fournier's gangrene or necrotizing fasciitis of the penis or scrotum directly resulting from masturbation. More frequently, occasional reports exist of male patients with other medical and surgical genital complications due to masturbation, autoerotic, and other sexual activities. Past published complications include direct bacterial inoculation or fat embolism after penile injection, and urethral tears and lodged foreign bodies in the bladder after urethral self-instrumentation for erotic stimulation [10–12]. Reports of penile incarceration injury after placement of constricting rings and ring-like devices exist and can rarely lead to Fournier's gangrene or penile necrosis [13–16].

In a classic account, Fournier himself did report that gangrene “could be seen as a consequence of pulling of the skin of the penis” and “as a result of violent twisting of the penis during erection” but also that he had never seen gangrene as a result of “excessive masturbation” [6]. Nearly 130 years later, complications from excessive masturbation have proven to be exceedingly rare, but, as this case illustrates, they can be life threatening.
